# Aqua­chlorido(3,5-dinitro-2-oxidobenzo­ato-κ^2^ *O* ^1^,*O* ^2^)(1,10-phenanthroline-κ^2^ *N*,*N*′)chromium(III)

**DOI:** 10.1107/S1600536812009324

**Published:** 2012-03-07

**Authors:** Zhao-Hui Meng, Hui Lian, Shu-Shen Zhang, Yu-Quan Feng

**Affiliations:** a College of Chemistry and Pharmacy Engineering, Nanyang Normal University, Nanyang 473061, People’s Republic of China; b Department of Anatomy, College of Basic Medical Science, Xinxiang Medical College, Xinxiang 453003, People’s Republic of China

## Abstract

In the title compound, [Cr(C_7_H_2_N_2_O_7_)Cl(C_12_H_8_N_2_)(H_2_O)], the Cr^III^ atom displays a distorted octa­hedral coordination geometry, with the chelating phenantroline and 3,5-dinitro­salicylate ligands in *trans* positions. In the crystal, mol­ecules are connected *via* O—H⋯O hydrogen bonds into a two-dimensional framework parallel to (100). In addition, there are π–π stacking inter­actions between phenanthroline ligands along the *c* axis, with a mean inter­planar distance of 3.456 (4) Å.

## Related literature

For the structure of a similar Mn^III^ complex, see: Tan & Tang (1996 ▶). For π –π stacking inter­actions in metal complexes, see: Janiak (2000 ▶).
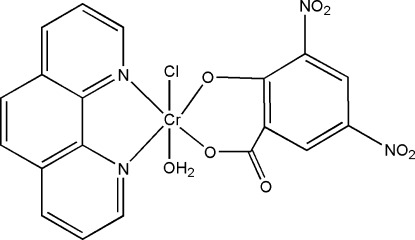


## Experimental

### 

#### Crystal data

[Cr(C_7_H_2_N_2_O_7_)Cl(C_12_H_8_N_2_)(H_2_O)]
*M* *_r_* = 511.78
Monoclinic, 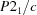

*a* = 13.868 (7) Å
*b* = 16.158 (8) Å
*c* = 9.348 (5) Å
β = 105.947 (9)°
*V* = 2014.2 (17) Å^3^
*Z* = 4
Mo *K*α radiation
μ = 0.76 mm^−1^
*T* = 296 K
0.19 × 0.15 × 0.13 mm

#### Data collection

Bruker APEXII CCD diffractometer
Absorption correction: multi-scan (*SADABS*; Bruker, 1997 ▶) *T*_min_ = 0.869, *T*_max_ = 0.908
10867 measured reflections
3952 independent reflections
2378 reflections with *I* > 2σ(*I*)
*R*_int_ = 0.060

#### Refinement

*R*[*F* ^2^ > 2σ(*F* ^2^)] = 0.058
*wR*(*F* ^2^) = 0.161
*S* = 1.04
3952 reflections
306 parameters
2 restraints
H atoms treated by a mixture of independent and constrained refinement
Δρ_max_ = 0.48 e Å^−3^
Δρ_min_ = −0.49 e Å^−3^

### 

Data collection: *APEX2* (Bruker, 1997 ▶); cell refinement: *SAINT* (Bruker, 1997 ▶); data reduction: *SAINT*; program(s) used to solve structure: *SHELXS97* (Sheldrick, 2008 ▶); program(s) used to refine structure: *SHELXL97* (Sheldrick, 2008 ▶); molecular graphics: *SHELXTL* (Sheldrick, 2008 ▶); software used to prepare material for publication: *SHELXTL*.

## Supplementary Material

Crystal structure: contains datablock(s) I, global. DOI: 10.1107/S1600536812009324/gk2465sup1.cif

Structure factors: contains datablock(s) I. DOI: 10.1107/S1600536812009324/gk2465Isup2.hkl

Additional supplementary materials:  crystallographic information; 3D view; checkCIF report

## Figures and Tables

**Table 1:** Selected bond lengths (Å)

Cr1—O3	1.906 (3)
Cr1—O1	1.926 (3)
Cr1—O8	2.017 (4)
Cr1—N1	2.056 (4)
Cr1—N2	2.065 (3)
Cr1—Cl1	2.2705 (17)

**Table 2:** Hydrogen-bond geometry (Å, °)

*D*—H⋯*A*	*D*—H	H⋯*A*	*D*⋯*A*	*D*—H⋯*A*
O8—H1*WB*⋯O6^i^	0.83 (2)	1.94 (2)	2.759 (5)	168 (5)
O8—H1*WA*⋯O2^ii^	0.81 (2)	1.81 (3)	2.581 (4)	160 (5)

Symmetry codes: (i) 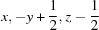; (ii) 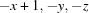.

**Fig. 1.**
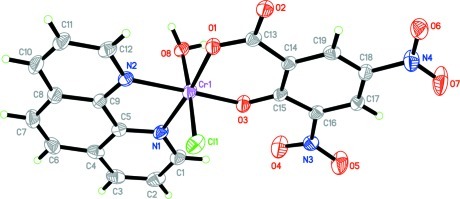
View of the title molecule with displacement ellipsoids drawn at the 30% probability level.

**Fig. 2.**
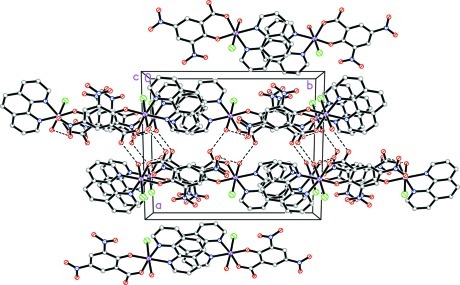
Crystal packing along the c axis. Hydrogen bonds are shown as dashed lines.

## supplementary crystallographic information

### Comment

Herein we report a mononuclear chromium(III) coordination compound [Cr(C_12_H_8_N_2_)(C_7_H_2_N_2_O_7_)Cl(H_2_O)] (Fig. 1) obtained with the use of 3,5-dinitrosalicylic acid and 1,10-phenanthroline ligands. In the structure of title compound, the chromium atom is octahedrally coordinated by two N atoms from the phenanthroline ligand, two O atoms from the (C_7_H_2_N_2_O_7_)^2- ^anion, one Cl ion and one water molecule. Bond lengths to the metal center are given in Table 1. The molecules are connected *via* O—H···O hydrogen bonds resulting in the formation of a two-dimensional supermolecular structure (Fig. 2). Moreover, there are π — π stacking interactions between phenanthroline ligands along the *c* axis due to the fact that these aromatic groups of phenanthroline ligands are parallel with each other. Such π — π stacking interactions between aromatic groups are rather popular in coordination compounds. Hydrogen bonds and π —π stacking interactions play a crucial role in stability of the crystal structure.

### Experimental

All chemicals were of reagent grade quality obtained from commercial sources and used without further purification. The title compound was synthesized from a mixture of CrCl_3_.6H_2_O (0.80 g, 3 mmol), 3,5-dinitrosalicylic acid (0.68 g, 3 mmol) and 1, 10-phenanthroline (0.60 g, 3 mmol), NaOH (0.08 g, 2 mmol) and ethanol (20 mL) by hydrothermal reaction. The mixture was stirred for half an hour, and then transferred into a Teflon-lined stainless steel autoclave (50 mL) and treated at 160 °C for 3 days. After the mixture was slowly cooled to room temperature, green block crystals suitable for X-ray structure determination were obtained.

### Refinement

The H atoms bonded to C were positioned geometrically and refined using a riding model, with C—H = 0.93 Å and with *U*_iso_(H) = 1.2 times *U*_eq_(C). The H atoms bonded to O atoms were located from Fourier difference maps and refined with distance restraints of O8—H1WA = 0.83 (2) Å, and O8—H1WB = 0.83 (2) Å.

### Crystal data

**Table:**

[Cr(C_7_H_2_N_2_O_7_)Cl(C_12_H_8_N_2_)(H_2_O)]	*F*(000) = 1036
*M**_r_* = 511.78	*D*_x_ = 1.688 Mg m^−^^3^
Monoclinic, *P*2_1_/*c*	Mo *K*α radiation, λ = 0.71073 Å
Hall symbol: -P 2ybc	Cell parameters from 1361 reflections
*a* = 13.868 (7) Å	θ = 2.6–20.2°
*b* = 16.158 (8) Å	µ = 0.76 mm^−^^1^
*c* = 9.348 (5) Å	*T* = 296 K
β = 105.947 (9)°	Block, green
*V* = 2014.2 (17) Å^3^	0.19 × 0.15 × 0.13 mm
*Z* = 4	

### Data collection

**Table:**

Bruker APEXII CCD diffractometer	3952 independent reflections
Radiation source: fine-focus sealed tube	2378 reflections with *I* > 2σ(*I*)
Graphite monochromator	*R*_int_ = 0.060
φ and ω scans	θ_max_ = 26.0°, θ_min_ = 2.5°
Absorption correction: multi-scan (*SADABS*; Bruker, 1997)	*h* = −17→14
*T*_min_ = 0.869, *T*_max_ = 0.908	*k* = −19→18
10867 measured reflections	*l* = −10→11

### Refinement

**Table:**

Refinement on *F*^2^	Primary atom site location: structure-invariant direct methods
Least-squares matrix: full	Secondary atom site location: difference Fourier map
*R*[*F*^2^ > 2σ(*F*^2^)] = 0.058	Hydrogen site location: inferred from neighbouring sites
*wR*(*F*^2^) = 0.161	H atoms treated by a mixture of independent and constrained refinement
*S* = 1.04	*w* = 1/[σ^2^(*F*_o_^2^) + (0.0753*P*)^2^] where *P* = (*F*_o_^2^ + 2*F*_c_^2^)/3
3952 reflections	(Δ/σ)_max_ < 0.001
306 parameters	Δρ_max_ = 0.48 e Å^−^^3^
2 restraints	Δρ_min_ = −0.49 e Å^−^^3^

### Special details

**Table:**

Geometry. All esds (except the esd in the dihedral angle between two l.s. planes) are estimated using the full covariance matrix. The cell esds are taken into account individually in the estimation of esds in distances, angles and torsion angles; correlations between esds in cell parameters are only used when they are defined by crystal symmetry. An approximate (isotropic) treatment of cell esds is used for estimating esds involving l.s. planes.

### Fractional atomic coordinates and isotropic or equivalent isotropic displacement parameters (Å^2^)

**Table:**

	*x*	*y*	*z*	*U*_iso_*/*U*_eq_	
Cr1	0.71896 (5)	−0.01477 (4)	0.02803 (8)	0.0398 (2)	
C1	0.8895 (3)	−0.0499 (3)	−0.1138 (5)	0.0486 (12)	
H1A	0.8952	0.0071	−0.1228	0.058*	
C2	0.9520 (3)	−0.1014 (3)	−0.1671 (5)	0.0549 (13)	
H2A	0.9985	−0.0790	−0.2113	0.066*	
C3	0.9445 (3)	−0.1854 (3)	−0.1540 (5)	0.0541 (13)	
H3A	0.9861	−0.2203	−0.1892	0.065*	
C4	0.8750 (3)	−0.2183 (3)	−0.0885 (5)	0.0466 (12)	
C5	0.8148 (3)	−0.1623 (3)	−0.0363 (5)	0.0373 (10)	
C6	0.8586 (4)	−0.3049 (3)	−0.0718 (5)	0.0538 (13)	
H6A	0.8986	−0.3429	−0.1038	0.065*	
C7	0.7875 (4)	−0.3331 (3)	−0.0115 (5)	0.0531 (13)	
H7A	0.7769	−0.3898	−0.0076	0.064*	
C8	0.7280 (4)	−0.2774 (3)	0.0467 (5)	0.0451 (12)	
C9	0.7416 (3)	−0.1913 (2)	0.0324 (5)	0.0372 (10)	
C10	0.6547 (4)	−0.3003 (3)	0.1147 (5)	0.0540 (14)	
H10A	0.6413	−0.3562	0.1241	0.065*	
C11	0.6019 (4)	−0.2423 (3)	0.1680 (5)	0.0504 (12)	
H11A	0.5542	−0.2584	0.2156	0.060*	
C12	0.6203 (3)	−0.1593 (3)	0.1502 (5)	0.0463 (11)	
H12A	0.5840	−0.1201	0.1866	0.056*	
C13	0.5958 (3)	0.1109 (3)	0.1121 (5)	0.0381 (10)	
C14	0.6519 (3)	0.1771 (2)	0.0562 (4)	0.0350 (10)	
C15	0.7266 (3)	0.1605 (2)	−0.0202 (5)	0.0346 (10)	
C16	0.7719 (3)	0.2324 (3)	−0.0637 (5)	0.0372 (10)	
C17	0.7469 (3)	0.3115 (3)	−0.0374 (5)	0.0403 (10)	
H17A	0.7791	0.3564	−0.0662	0.048*	
C18	0.6729 (3)	0.3229 (2)	0.0326 (5)	0.0413 (11)	
C19	0.6267 (3)	0.2570 (3)	0.0788 (5)	0.0398 (10)	
H19A	0.5772	0.2669	0.1265	0.048*	
Cl1	0.83175 (9)	0.00500 (7)	0.25272 (14)	0.0564 (4)	
N1	0.8216 (3)	−0.0795 (2)	−0.0502 (4)	0.0402 (9)	
N2	0.6876 (3)	−0.1336 (2)	0.0834 (4)	0.0377 (8)	
N3	0.8523 (3)	0.2233 (2)	−0.1369 (4)	0.0443 (9)	
N4	0.6453 (3)	0.4060 (2)	0.0641 (5)	0.0579 (11)	
O1	0.6129 (2)	0.03496 (17)	0.0950 (3)	0.0468 (8)	
O2	0.5312 (2)	0.13290 (17)	0.1731 (3)	0.0468 (8)	
O3	0.7505 (2)	0.08735 (17)	−0.0508 (3)	0.0462 (8)	
O4	0.8663 (3)	0.1591 (2)	−0.1910 (5)	0.0756 (12)	
O5	0.9030 (3)	0.2830 (2)	−0.1388 (6)	0.0961 (16)	
O6	0.5745 (3)	0.4138 (2)	0.1213 (4)	0.0697 (11)	
O7	0.6920 (4)	0.4640 (2)	0.0366 (5)	0.0934 (16)	
O8	0.6198 (2)	−0.0388 (2)	−0.1700 (4)	0.0450 (8)	
H1WB	0.608 (3)	0.0037 (19)	−0.222 (4)	0.050 (15)*	
H1WA	0.565 (2)	−0.059 (3)	−0.180 (5)	0.060 (17)*	

### Atomic displacement parameters (Å^2^)

**Table:**

	*U*^11^	*U*^22^	*U*^33^	*U*^12^	*U*^13^	*U*^23^
Cr1	0.0387 (4)	0.0349 (4)	0.0546 (5)	−0.0041 (3)	0.0277 (3)	−0.0005 (3)
C1	0.040 (3)	0.050 (3)	0.063 (3)	−0.007 (2)	0.027 (2)	−0.002 (2)
C2	0.039 (3)	0.074 (4)	0.061 (3)	−0.006 (2)	0.029 (3)	−0.007 (3)
C3	0.039 (3)	0.070 (4)	0.057 (3)	0.010 (3)	0.020 (2)	−0.010 (3)
C4	0.036 (3)	0.056 (3)	0.047 (3)	0.006 (2)	0.010 (2)	−0.007 (2)
C5	0.034 (2)	0.042 (2)	0.036 (3)	−0.0025 (19)	0.0100 (19)	−0.0016 (19)
C6	0.055 (3)	0.045 (3)	0.058 (3)	0.011 (2)	0.010 (3)	−0.006 (2)
C7	0.065 (3)	0.035 (3)	0.055 (3)	0.002 (2)	0.009 (3)	−0.003 (2)
C8	0.048 (3)	0.042 (3)	0.040 (3)	−0.004 (2)	0.003 (2)	0.004 (2)
C9	0.037 (3)	0.037 (2)	0.036 (2)	−0.0067 (19)	0.007 (2)	0.0013 (19)
C10	0.057 (3)	0.046 (3)	0.051 (3)	−0.018 (3)	0.003 (3)	0.013 (2)
C11	0.049 (3)	0.056 (3)	0.047 (3)	−0.016 (2)	0.015 (2)	0.012 (2)
C12	0.042 (3)	0.057 (3)	0.044 (3)	−0.009 (2)	0.018 (2)	0.004 (2)
C13	0.035 (2)	0.042 (3)	0.041 (3)	−0.004 (2)	0.017 (2)	−0.001 (2)
C14	0.034 (2)	0.037 (2)	0.037 (3)	−0.0049 (19)	0.0147 (19)	−0.0028 (18)
C15	0.030 (2)	0.037 (2)	0.038 (3)	−0.0050 (19)	0.0117 (18)	0.0013 (18)
C16	0.035 (2)	0.041 (2)	0.040 (3)	−0.0064 (19)	0.018 (2)	−0.0002 (18)
C17	0.041 (3)	0.035 (2)	0.047 (3)	−0.005 (2)	0.015 (2)	0.0027 (19)
C18	0.045 (3)	0.034 (2)	0.047 (3)	−0.004 (2)	0.016 (2)	−0.003 (2)
C19	0.033 (2)	0.050 (3)	0.039 (3)	−0.001 (2)	0.015 (2)	−0.006 (2)
Cl1	0.0573 (8)	0.0561 (7)	0.0591 (8)	−0.0148 (6)	0.0218 (6)	−0.0049 (6)
N1	0.036 (2)	0.042 (2)	0.047 (2)	−0.0067 (16)	0.0185 (18)	−0.0006 (16)
N2	0.035 (2)	0.040 (2)	0.042 (2)	−0.0020 (16)	0.0181 (17)	0.0044 (16)
N3	0.040 (2)	0.047 (2)	0.052 (3)	−0.0023 (19)	0.0230 (19)	0.0071 (19)
N4	0.075 (3)	0.040 (2)	0.065 (3)	−0.001 (2)	0.030 (3)	−0.007 (2)
O1	0.0442 (18)	0.0342 (17)	0.076 (2)	−0.0014 (14)	0.0400 (17)	0.0005 (15)
O2	0.0453 (19)	0.0435 (17)	0.064 (2)	−0.0039 (14)	0.0364 (17)	−0.0067 (15)
O3	0.0492 (19)	0.0367 (17)	0.066 (2)	−0.0037 (14)	0.0385 (17)	−0.0027 (14)
O4	0.088 (3)	0.056 (2)	0.113 (3)	−0.011 (2)	0.078 (3)	−0.013 (2)
O5	0.090 (3)	0.052 (2)	0.182 (5)	−0.014 (2)	0.096 (3)	0.006 (3)
O6	0.070 (3)	0.050 (2)	0.103 (3)	−0.0038 (18)	0.048 (2)	−0.0191 (19)
O7	0.136 (4)	0.0358 (19)	0.144 (4)	−0.017 (2)	0.098 (3)	−0.008 (2)
O8	0.040 (2)	0.0435 (19)	0.058 (2)	−0.0070 (16)	0.0245 (17)	0.0070 (16)

### Geometric parameters (Å, º)

**Table:**

Cr1—O3	1.906 (3)	C10—H10A	0.9300
Cr1—O1	1.926 (3)	C11—C12	1.383 (6)
Cr1—O8	2.017 (4)	C11—H11A	0.9300
Cr1—N1	2.056 (4)	C12—N2	1.325 (5)
Cr1—N2	2.065 (3)	C12—H12A	0.9300
Cr1—Cl1	2.2705 (17)	C13—O2	1.239 (5)
C1—N1	1.332 (5)	C13—O1	1.268 (5)
C1—C2	1.389 (6)	C13—C14	1.498 (6)
C1—H1A	0.9300	C14—C19	1.369 (5)
C2—C3	1.370 (7)	C14—C15	1.437 (6)
C2—H2A	0.9300	C15—O3	1.281 (5)
C3—C4	1.382 (7)	C15—C16	1.431 (5)
C3—H3A	0.9300	C16—C17	1.364 (6)
C4—C5	1.406 (6)	C16—N3	1.467 (5)
C4—C6	1.434 (6)	C17—C18	1.372 (6)
C5—N1	1.350 (5)	C17—H17A	0.9300
C5—C9	1.422 (6)	C18—C19	1.372 (6)
C6—C7	1.343 (7)	C18—N4	1.448 (5)
C6—H6A	0.9300	C19—H19A	0.9300
C7—C8	1.425 (7)	N3—O4	1.193 (4)
C7—H7A	0.9300	N3—O5	1.197 (5)
C8—C10	1.389 (7)	N4—O7	1.207 (5)
C8—C9	1.415 (6)	N4—O6	1.247 (5)
C9—N2	1.363 (5)	O8—H1WB	0.832 (19)
C10—C11	1.364 (7)	O8—H1WA	0.810 (19)
			
O3—Cr1—O1	92.39 (12)	C10—C11—C12	119.1 (5)
O3—Cr1—O8	89.04 (14)	C10—C11—H11A	120.4
O1—Cr1—O8	89.40 (14)	C12—C11—H11A	120.4
O3—Cr1—N1	92.74 (13)	N2—C12—C11	122.6 (5)
O1—Cr1—N1	173.41 (13)	N2—C12—H12A	118.7
O8—Cr1—N1	86.54 (14)	C11—C12—H12A	118.7
O3—Cr1—N2	171.05 (13)	O2—C13—O1	121.3 (4)
O1—Cr1—N2	94.24 (13)	O2—C13—C14	117.8 (4)
O8—Cr1—N2	85.03 (14)	O1—C13—C14	120.9 (4)
N1—Cr1—N2	80.24 (14)	C19—C14—C15	120.1 (4)
O3—Cr1—Cl1	93.60 (11)	C19—C14—C13	116.2 (4)
O1—Cr1—Cl1	91.96 (11)	C15—C14—C13	123.7 (4)
O8—Cr1—Cl1	176.97 (11)	O3—C15—C16	121.7 (4)
N1—Cr1—Cl1	91.85 (11)	O3—C15—C14	123.3 (4)
N2—Cr1—Cl1	92.18 (10)	C16—C15—C14	115.0 (4)
N1—C1—C2	122.1 (5)	C17—C16—C15	123.8 (4)
N1—C1—H1A	118.9	C17—C16—N3	116.2 (4)
C2—C1—H1A	118.9	C15—C16—N3	120.0 (4)
C3—C2—C1	119.5 (5)	C16—C17—C18	118.2 (4)
C3—C2—H2A	120.3	C16—C17—H17A	120.9
C1—C2—H2A	120.3	C18—C17—H17A	120.9
C2—C3—C4	120.0 (4)	C19—C18—C17	121.3 (4)
C2—C3—H3A	120.0	C19—C18—N4	118.9 (4)
C4—C3—H3A	120.0	C17—C18—N4	119.8 (4)
C3—C4—C5	117.3 (4)	C14—C19—C18	121.5 (4)
C3—C4—C6	125.1 (5)	C14—C19—H19A	119.2
C5—C4—C6	117.6 (4)	C18—C19—H19A	119.2
N1—C5—C4	122.7 (4)	C1—N1—C5	118.3 (4)
N1—C5—C9	116.6 (4)	C1—N1—Cr1	128.3 (3)
C4—C5—C9	120.7 (4)	C5—N1—Cr1	113.4 (3)
C7—C6—C4	122.2 (5)	C12—N2—C9	118.4 (4)
C7—C6—H6A	118.9	C12—N2—Cr1	129.4 (3)
C4—C6—H6A	118.9	C9—N2—Cr1	112.0 (3)
C6—C7—C8	121.1 (4)	O4—N3—O5	121.9 (4)
C6—C7—H7A	119.5	O4—N3—C16	121.2 (4)
C8—C7—H7A	119.5	O5—N3—C16	116.9 (4)
C10—C8—C9	116.0 (4)	O7—N4—O6	122.9 (4)
C10—C8—C7	125.4 (4)	O7—N4—C18	119.3 (4)
C9—C8—C7	118.5 (4)	O6—N4—C18	117.7 (4)
N2—C9—C8	122.7 (4)	C13—O1—Cr1	129.1 (3)
N2—C9—C5	117.5 (4)	C15—O3—Cr1	127.7 (3)
C8—C9—C5	119.8 (4)	Cr1—O8—H1WB	111 (3)
C11—C10—C8	121.2 (4)	Cr1—O8—H1WA	125 (3)
C11—C10—H10A	119.4	H1WB—O8—H1WA	103 (5)
C8—C10—H10A	119.4		

### Hydrogen-bond geometry (Å, º)

**Table:**

*D*—H···*A*	*D*—H	H···*A*	*D*···*A*	*D*—H···*A*
O8—H1*WB*···O6^i^	0.83 (2)	1.94 (2)	2.759 (5)	168 (5)
O8—H1*WA*···O2^ii^	0.81 (2)	1.81 (3)	2.581 (4)	160 (5)

Symmetry codes: (i) *x*, −*y*+1/2, *z*−1/2; (ii) −*x*+1, −*y*, −*z*.
